# Why Should Nurses Engage in Artificial Intelligence Research?

**DOI:** 10.1177/23779608251406553

**Published:** 2025-12-17

**Authors:** Abdulqadir J. Nashwan

**Affiliations:** Nursing & Midwifery Research Department (NMRD), Hamad Medical Corporation, Doha, Qatar

**Keywords:** nursing, artificial intelligence, nursing research, patient care

## Abstract

This letter highlights the necessity of nursing engagement in artificial intelligence (AI) research and its potential impact on healthcare. As AI transforms patient care, diagnostics, and operational efficiency, it is crucial for nurses, the largest healthcare workforce, to engage in AI development actively. Nurses’ experiential knowledge and ethical perspectives can enhance AI tools, ensuring they are patient-centered and practically applicable. This letter underscores the importance of interdisciplinary collaboration and advocates for increased nursing involvement in AI research to achieve better healthcare outcomes.

Dear Editor,

Artificial intelligence (AI) has rapidly evolved from a futuristic concept to a pervasive force reshaping industries worldwide (Mossavar-Rahmani & Zohuri, 2024). AI is transforming patient care, diagnostics, treatment planning, and operational efficiency (Khalifa & Albadawy, 2024). From predictive analytics and personalized medicine to robotic surgeries and virtual health assistants, AI's integration into healthcare promises improved outcomes, enhanced patient experiences, and reduced costs (Olawade et al., 2024). As the field grows, it becomes crucial for diverse stakeholders to contribute to AI research and development to ensure alignment with the multifaceted needs of healthcare systems. Among these stakeholders, nurses stand out as an essential but often underrepresented group.

Investment in AI is significant across various industries, with healthcare, finance, and automotive sectors leading the way. Healthcare AI investments focus on diagnostics, personalized medicine, and operational efficiencies, with substantial growth expected beyond the $6.6 billion mark noted in 2021 (Lin & Alvarez, 2021). For instance, finance utilizes AI for fraud detection and risk management, seeing around $10 billion in annual investments (Sabharwal, 2018). Retail and manufacturing also invest heavily in AI to enhance customer experience and optimize operations, with annual investments of around $5 billion and $4 billion, respectively (Bughin et al., 2017).

Nurses represent the largest segment of the healthcare workforce, providing frontline care and an intimate understanding of patient needs and healthcare delivery processes (Smiley et al., 2021). This unique position offers nurses invaluable insights into AI applications and innovations. The urgent need for nurses to be involved in the early stages of technology design, acquisition, and implementation cannot be overstated (Dykes & Chu, 2021; Rony et al., 2024). It is time to change this narrative and recognize the significant benefits that can arise from active nursing engagement in AI research.

Firstly, nurses possess a wealth of experiential knowledge and practical expertise that can inform AI development. Their daily interactions with patients give them a deep-rooted understanding of their behaviors, preferences, and needs. This insight is crucial for developing AI tools that are clinically effective, user-friendly, and aligned with patient care practices.

Secondly, nurses can play a pivotal role in addressing the ethical considerations surrounding AI in healthcare (Nashwan & Abujaber, 2023). Issues such as patient privacy, data security, and the ethical use of AI in decision-making processes require careful deliberation. Nurses can contribute significantly to these discussions with their commitment to patient advocacy and ethical practice. Their participation ensures that AI technologies uphold the highest standards of patient care and respect for individual rights.

Furthermore, nursing-led AI research can drive innovations tailored to improve nursing practice and patient outcomes (Rony et al., 2024). For instance, AI can assist with workload management, predicting patient deterioration, and enhancing care coordination. As end-users of these technologies, nurses can provide essential feedback to refine these tools, making them more effective and practical. Their involvement can also foster the development of AI-driven educational tools, enhancing nursing training and continuing education.

Lastly, engaging nurses in AI research promotes interdisciplinary collaboration, a cornerstone of effective healthcare innovation. It encourages a holistic approach that integrates insights from various healthcare professionals, leading to more robust, comprehensive AI solutions. This collaboration can also help demystify AI for nursing professionals, fostering a culture of technological proficiency and openness to innovation within the nursing community.

To conclude, the integration of AI in healthcare presents unprecedented opportunities to revolutionize patient care and operational efficiency. However, to realize AI's full potential, it is imperative to involve nurses actively in AI research and development. As the largest and most influential segment of the healthcare workforce, nurses bring unique perspectives, ethical considerations, and practical insights that can significantly enhance AI technologies. Their engagement ensures that AI innovations are patient-centered, ethically sound, and practically applicable, ultimately leading to better healthcare outcomes and a more efficient healthcare system.
